# Giant mediastinal teratoma mimicking pericardial cyst: A case report with brief review of the literature

**DOI:** 10.3892/mi.2025.262

**Published:** 2025-08-19

**Authors:** Fahmi H. Kakamad, Marwan N. Hassan, Rebaz M. Ali, Hawkar A. Nasralla, Suhaib H. Kakamad, Fakher Abdullah, Govand Nawzad Abubaker, Muhammed Sherzad Ezzat, Sozy Amanj Kareem, Berun A. Abdalla

**Affiliations:** 1 Department of Scientific Affairs, Smart Health Tower, Sulaymaniyah 46001, Iraq; 2 Kscien Organization for Scientific Research (Middle East office), Sulaymaniyah 46001, Iraq; 3 College of Medicine, University of Sulaimani, Sulaymaniyah 46001, Iraq

**Keywords:** teratoma, thoracoscopy, thoracotomy, sternotomy, video-assisted thoracoscopy

## Abstract

Giant teratomas with bilateral mediastinal extension are rare. The present case report highlights the challenging resection of a giant anterior mediastinal cystic teratoma extending bilaterally using video-assisted thoracoscopic surgery (VATS), rendering it one of the largest mediastinal teratomas managed with VATS in the current literature. A 47-year-old woman presented with a 1-year history of central chest pain. The physical examination did not reveal any notable findings. A computed tomography coronary angiogram revealed a thin-walled cyst in the anterior mediastinum, measuring ~12x10x9 cm, consistent with a pericardial cyst. The cyst was completely resected using a bilateral thoracoscopic approach, and the patient was transferred to recovery in stable condition. A histopathological examination confirmed a mature cystic teratoma. In addition, a brief literature review conducted using the PubMed and Google Scholar databases identified 11 case reports of mediastinal teratomas resected using VATS. The mass sizes ranged from 4x4 to 17x10 cm. The majority of patients in these case reports were female (81.81%), with an average age of 21.45±12.69 years, ranging from 4 to 53 years. Notably, 63.63% of these patients were asymptomatic. On the whole, the present study demonstrates that large mediastinal cysts can be resected using VATS, which can provide a safe and effective alternative to conventional open thoracotomy or sternotomy. This approach may reduce surgical trauma and support faster post-operative recovery.

## Introduction

A teratoma is a germ cell tumor that can develop in gonadal and extragonadal locations (1). These tumors are most commonly found in the sacrococcygeal region, although they can also occur in the ovaries, head and neck, mediastinum, testes and central nervous system (2). Teratomas are composed of tissues from embryonic germ layers: Endoderm, mesoderm and ectoderm. They account for <15% of anterior mediastinal masses (1).

Primary mediastinal teratomas are rare, primarily affect women, and are usually located in the upper middle anterior mediastinum. Of note, ~80% of these teratomas are benign (3). Traditionally, sternotomy has been the standard method for resecting anterior mediastinal masses, as it provides sufficient exposure for easy removal (1). However, due to the morbidity associated with sternotomy, surgeons have increasingly explored video-assisted thoracoscopic surgery (VATS) to expand its applications (1).

VATS provides a magnified view of the operative field, enabling precise dissection, while minimizing trauma to surrounding structures (4,5). Despite its increasing use, VATS has traditionally been reserved for smaller tumors due to concerns about limited visualization and difficulty in manipulating large or adherent masses through small ports. Nevertheless, recent studies and case reports have demonstrated the feasibility and safety of using VATS even for the resection of giant mature teratomas in the mediastinum, challenging earlier size-based limitations with increasing experience and improved instrumentation (2,3,4).

Bilateral VATS is considered for mediastinal tumors that extend across the midline or involve both hemithoraces, where complete resection from a single side is challenging (6). By contrast, very large tumors or those with marked invasion into vital structures, such as the great vessels or pericardium, may necessitate open surgery to ensure adequate exposure and safety (7). Compared to traditional open approaches, bilateral VATS provides reduced surgical trauma, reduced post-operative pain, a more rapid recovery and comparable oncological outcomes in appropriately selected cases (6,7).

The present study describes a rare case of a giant mature cystic teratoma with bilateral mediastinal extension that was successfully resected using a bilateral thoracoscopic approach. The present study also includes a brief review of the literature in an aim to contextualize the findings within the scope of current minimally invasive thoracic surgery practices. The present study adheres to the CaReL Guidelines, with all references thoroughly assessed for eligibility (8,9).

## Case report

### Patient information

A 47-year-old woman with a 1-year history of central chest pain presented to Smart Health Tower (Sulaymaniyah, Iraq). She had visited the emergency department on multiple occasions, where repeated cardiac assessments, including electrocardiograms and troponin levels, consistently revealed nothing out of the ordinary. The last cardiology evaluation included an echocardiogram and a computed tomography (CT) coronary angiogram (CTCA). The echocardiogram was normal, while the CTCA revealed a large mediastinal mass. She was subsequently referred to the cardiothoracic surgery clinic. At the time of referral, she continued to report central chest pain without radiation or other associated symptoms. Her medical history included hypertension. She was a non-smoker with no prior surgical history.

### Clinical findings

Upon examination, there was no chest tenderness. Cardiac auscultation revealed normal heart sounds without murmurs. The bilateral air entry was clear with no additional sounds. Her vital signs were all within normal limits.

### Diagnostic approach

**Figure 1:**
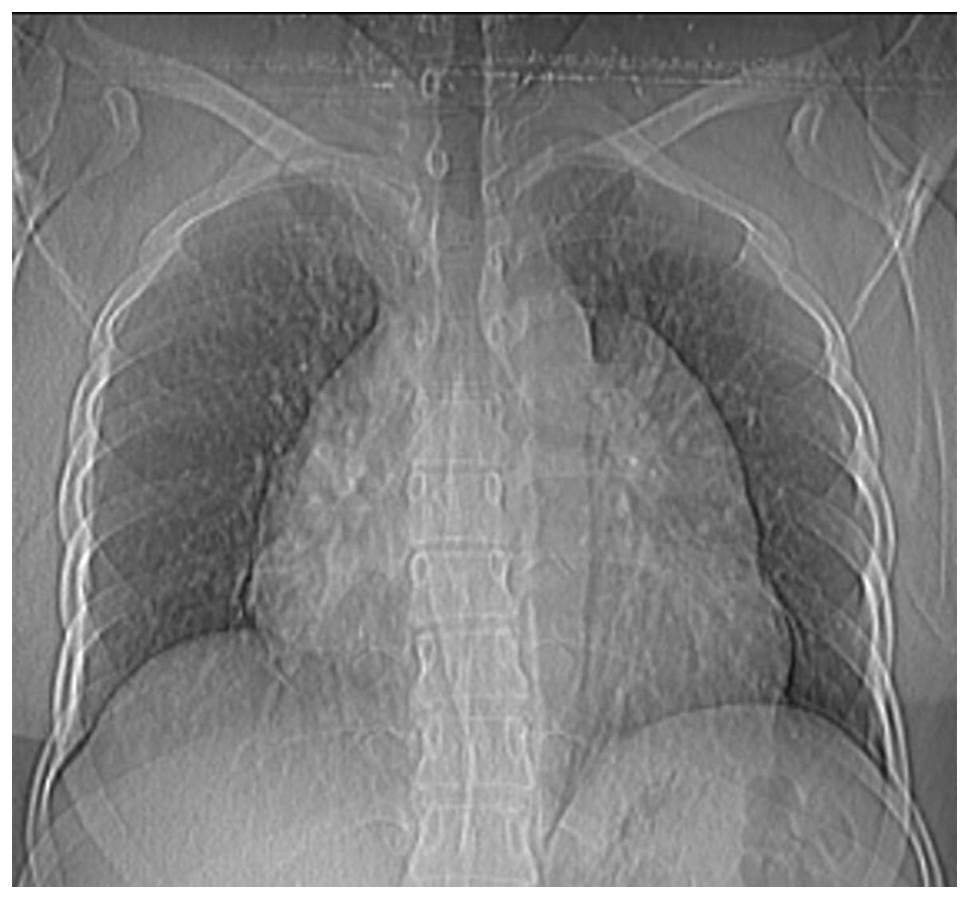
Chest X-ray illustrating an enlarged mediastinum.

**Figure 2:**
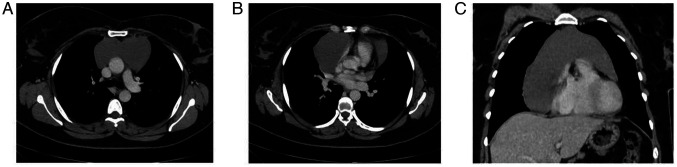
A contrast-enhanced chest computed tomography scan illustrating the following: (A) A large, thin-walled cystic lesion in the anterior mediastinum. (B) The cyst extending into both the right and left sides of the mediastinum, with a greater extension towards the right. (C) On the right side, the cyst extends further down to the level of the diaphragm.

The chest X-ray (CXR) revealed an enlarged mediastinum (Fig. 1). A CTCA was performed, which confirmed the presence of a thin-walled cyst in the anterior mediastinum, measuring ~12x10x9 cm. The cyst was in contact with the pericardium and extended to both the right and left sides of the mediastinum. The imaging findings suggested a benign cyst, most likely a pericardial cyst (Fig. 2).

### Therapeutic interventions

Given the size and bilateral location of the cyst, with a greater extension on the right side, a bilateral thoracoscopic approach for mediastinal cyst resection was planned. Under general anesthesia, a double-lumen endotracheal tube was inserted, and the patient was placed in a 30-45˚ semi-supine position with arms extended to allow access to both sides of the chest.

**Figure 3:**
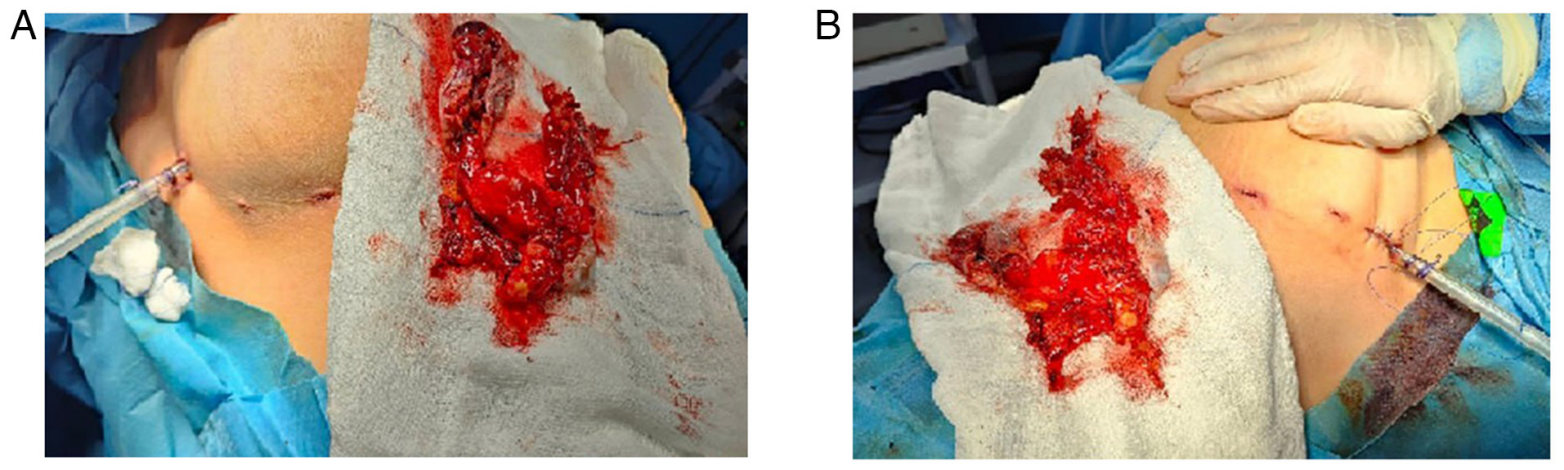
Bilateral thoracic access with the excised cyst wall: (A) Right side view, (B) left side view.

**Figure 4:**
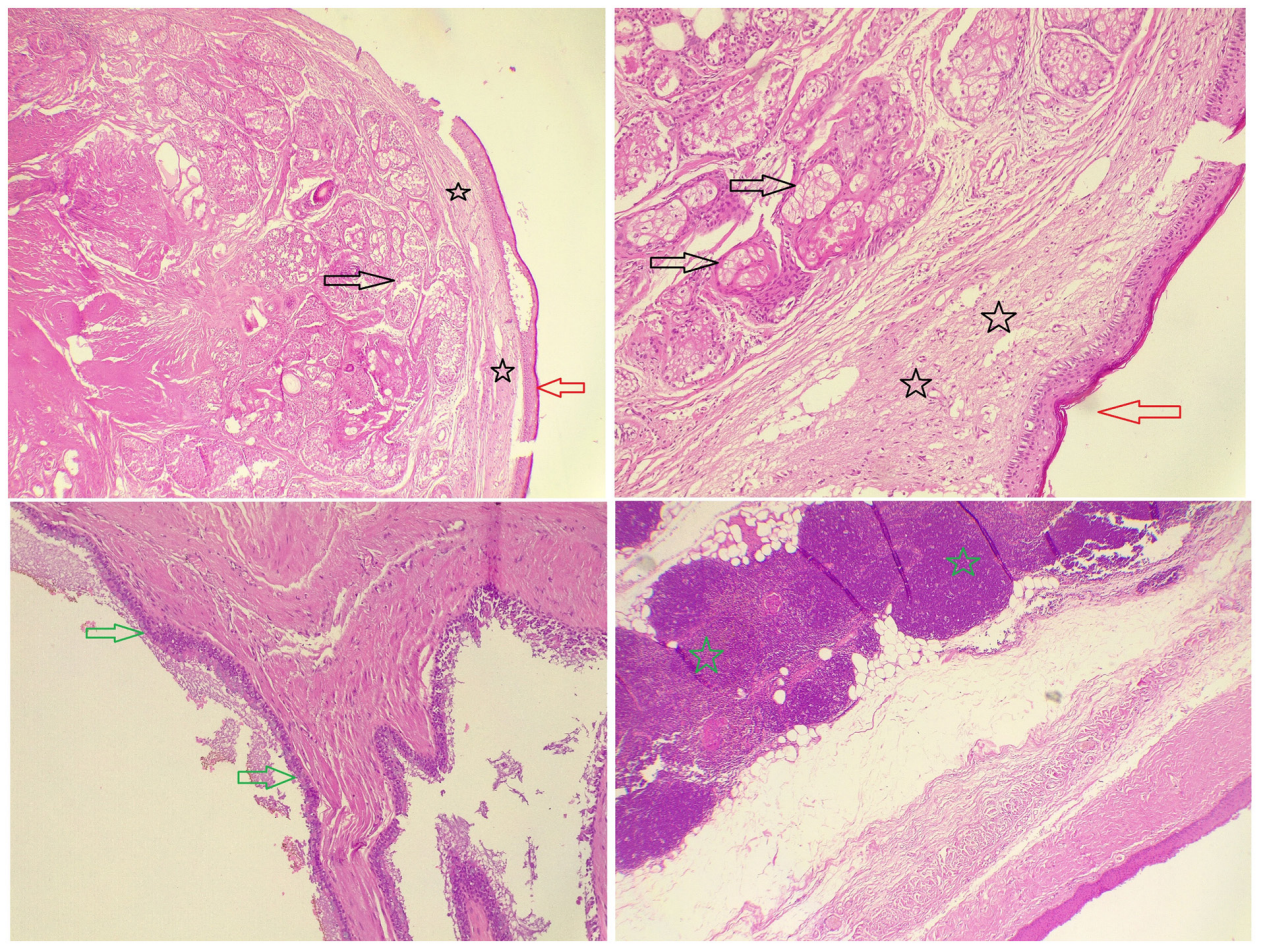
Multiple hematoxylin and eosin-stained sections, demonstrating two types of epithelial cell lining, stratified squamous epithelial cells (red arrows) and ciliated columnar epithelial cells (green arrows), with the presence of glial tissue (black stars) and sebaceous glands (black arrows), with unremarkable thymic tissue (green stars) surrounded by adipose tissue.

The right lung was deflated to provide a clear operative field. A standard three-port technique was used: A 10-mm camera port and two 5-mm working ports were inserted. A thorough inspection of the mediastinum revealed a large cystic lesion in the anterior mediastinum, extending bilaterally. The cyst was carefully separated from surrounding mediastinal structures, including the pericardium and great vessels, using a combination of blunt dissection and electrocautery. During dissection, the cyst was inadvertently ruptured, releasing thick and dark green fluid. The fluid was immediately aspirated, and care was taken to minimize the contamination of the surrounding thoracic cavity. The cyst was fully mobilized while preserving vital structures. Once mobilized on the right, the same procedure was performed on the left side. The cyst was freed from the mediastinal attachments bilaterally. It was then placed in an endoscopic retrieval bag, and the 10-mm port site was slightly enlarged to facilitate removal. Bilateral chest tubes were placed through the 10-mm ports for drainage. The procedure was completed using the bilateral approach without the need for conversion to open surgery (Fig. 3). The patient was transferred to recovery in a stable condition. A histopathological examination was performed by the authors' laboratory. The 5-µm-thick sections were fixed in 10% neutral-buffered formalin at room temperature for 24 h and embedded in paraffin. They were subsequently stained with hematoxylin and eosin (Bio Optica Co.) for 1-2 min at room temperature, and then examined under a light microscope (Leica Microsystems GmbH). The histopathological examination revealed a multiloculated, thick fibro-muscular wall, lined predominantly by stratified squamous cells, with focal areas containing ciliated columnar epithelial cells and goblet cells. Benign skin appendages, including sebaceous glands and sweat glands, were also present, confirming a mature cystic teratoma with no malignant components (Fig. 4).

### Follow-up and outcomes

The chest tube was non-functional on the second post-operative day, and a follow-up CXR revealed complete lung expansion (images not available). Both chest tubes were subsequently removed. The patient was discharged home in stable condition 48 h following surgery. After 10 days, the patient returned for a follow-up visit. A clinical examination revealed good bilateral air entry without abnormal sounds. A follow-up CXR revealed normal lung expansion with no residual air.

## Discussion

Various mediastinal masses or cysts can be distinguished based on their anatomical location. In the anterior mediastinum, the most common tumors include thymomas (31%), lymphomas (23%), and germ cell tumors (17%). Among the germ cell tumors in this region, mature teratomas make up 60% (10).

Mediastinal teratomas represent 5-10% of all mediastinal tumors. They arise from stem cells with vascular potential during the development of thymic tissue in embryonic life and contain multiple germ layers (2). Mediastinal mature teratomas are commonly found in young adults, with 50-62% of cases being asymptomatic. Conversely, 36-41% of patients experience symptoms related to tumor perforation, including chest pain, fever, hemoptysis and expectoration of the tumor contents (11). In the case in the present study, the patient had central chest pain for 1 year, even though the cyst remained intact.

While a CXR is frequently the first diagnostic tool for mediastinal teratomas, a chest CT scan is considered the primary diagnostic method (2). In the present case report, a CXR indicated an enlarged mediastinum, and a CTCA identified a large, thin-walled cyst in the anterior mediastinum.

In the past, open thoracotomy and sternotomy were the primary approaches for treating thoracic disorders. However, with the rapid advancement of minimally invasive surgery, VATS is now commonly used to diagnose and treat various chest-related conditions (12). Mouroux *et al* (13) reported the first mediastinal cyst excision using the VATS technique in 1991. Since then, results of mediastinal cyst excisions via the thoracoscopic approach have been documented primarily as case reports or brief case series.

The minimally invasive treatment of mediastinal teratomas has notable advantages, with complete thoracoscopic surgery being the treatment of choice. However, there are limited reports on the use of complete thoracoscopic resection for intrathoracic masses >50 mm in diameter (3). A brief literature review using PubMed and Google Scholar identified 11 case reports of mediastinal teratomas resected using VATS (2,3,10,11,14-20). Of these cases, 9 patients were female (81.81%), with a mean age of 21.45±12.69 years, ranging from 4 to 53 years. Among these patients, 63.63% were asymptomatic. The earliest case was reported by Furukawa *et al* (20) in 1994, who successfully resected a 7x5 cm mediastinal cystic teratoma. The most recent case, reported by Demir *et al* (2), involved the removal of a 7.5x3.6 cm teratoma. The largest resected teratoma, measuring 17x10 cm, was reported by Kuroda *et al* (15) in 2014 (Table I). The case presented herein involved a 12x10 cm mediastinal mature cystic teratoma, rendering it the third largest among the reported cases.

Shintani *et al* (21) conducted a retrospective study involving 15 patients with benign mediastinal mature teratomas who underwent thoracoscopic procedures. Of these patients, 60% were asymptomatic, with the lesions discovered incidentally on CXR images. The remaining patients presented with pre-operative symptoms, including chest pain, chest tightness, back pain, cough and fever. The mean tumor diameter was 5.3 cm, with the largest measuring 8.5 cm (21). However, in another study by Tsubochi *et al* (5), from 1998 to 2013, 13 patients with mediastinal mature teratomas underwent VATS. The tumor sizes ranged from 5 to 12 cm, with a mean size of 8 cm. None of the patients required conversion to an open procedure (5).

Bilateral VATS for mediastinal tumors remains relatively uncommon and is considered a novel technique in the thoracic surgery literature. The majority of published studies and reports focus on the unilateral VATS approach. Case reports and small series typically describe bilateral VATS in rare clinical scenarios, such as large tumors crossing the midline or requiring bilateral exposure (6,22). In a recent series involving 54 patients who underwent VATS for concurrent pulmonary and mediastinal lesions, only three cases utilized a bilateral approach, while the majority were managed unilaterally (22). To date, bilateral VATS performed for the resection of mature teratomas has not been documented. Among the 11 reviewed cases of mediastinal teratoma resection via VATS, none utilized a bilateral approach. By contrast, the case in the present study necessitated bilateral VATS due to the bilateral mediastinal extension of the tumor. To the best of our knowledge, this represents the first reported case of a giant mature teratoma successfully resected using a bilateral VATS approach.

A VATS resection for a mediastinal mature teratoma may sometimes be converted to an open procedure, typically due to the dense adhesions to surrounding structures of the tumor. Additionally, large tumors pose challenges during thoracoscopic procedures, as their size limits adequate exposure and manipulation (21). Cyst aspiration during thoracoscopy allows the operator to capture and manipulate the cyst easily. As experience with thoracoscopy has grown, the need to switch to thoracotomy has become increasingly rare. However, in cases of severe pleural adhesions, open thoracotomy becomes unavoidable (23). In the case described herein, the cyst was unintentionally ruptured during dissection, causing the release of thick, dark green fluid. The fluid was quickly aspirated, and precautions were implemented to avoid contaminating the surrounding thoracic cavity. The entire cyst was removed without the need for a thoracotomy.

When a malignant component is suspected, it is crucial to remove the tumor through an open procedure without exposing it. However, reports of malignant components coexisting in mature teratomas are rare (21). Chang *et al* (19) documented rapid tumor spread in a patient who underwent VATS resection of a teratoma with malignant transformation, where cyst rupture during surgery led to dissemination. While malignant transformation in teratomas is extremely rare, thorough and complete resection, including the cystic wall, is essential in cases of intact mediastinal mature teratomas to prevent the risk of relapse from a potentially malignant component.

In conclusion, large mediastinal cysts can be resected using VATS, which can provide a safe and effective alternative to conventional open thoracotomy or sternotomy. This approach may reduce surgical trauma and support a more rapid post-operative recovery.

## Figures and Tables

**Table I:** Reviewed cases identified in the literature of mediastinal teratoma resected via VATS.

First author	Year of publication	No. of cases	Age (years)	Sex	Clinical presentation	Diagnostic modalities	Type of the teratoma	Size of the mass (cm)	Location	VATS laterality	Post-operative course	(Refs.)
Demir	2024	1	13	Male	Persistent cough	X-ray, CT scan, MRI	Mature teratoma	7.5x3.6	The prevascular area of the anterior mediastinum	Unilateral (right)	Uneventful	(2)
Xiao-Dong	2019	1	28	Female	Chest pain	Echo, CT scan	Mature cystic teratoma	8.2x7.2	The left lateral of the mediastinum	Unilateral (left)	Uneventful	(3)
Cheng	2000	1	26	Male	Asymptomatic	CT scan	TMT	8x7	Anterior mediastinum	Unilateral (right)	Uneventful	(10)
Matsuoka	2018	1	15	Female	Asymptomatic	CT scan	Mature cystic teratoma	6.5x3.5	Mediastinal aspect	Unilateral (right)	Uneventful	(11)
Rothermel	2013	1	26	Female	Asymptomatic	X-ray, MRI, Echo	Mature cystic teratoma	10.8x9.4	Inferior-anterior mediastinum	Unilateral (right)	Uneventful	(14)
Kuroda	2014	1	16	Female	Chest pain	CT scan	Mature cystic teratoma	17x10	Anterior mediastinum	Unilateral (right)	Uneventful	(15)
Miyauchi	2014	1	15	Female	Asymptomatic	CT scan	Mature cystic teratoma	16x14	Anterior mediastinum	Unilateral (left)	Uneventful	(16)
Codrich	2012	1	4	Female	Asymptomatic	X-ray, MRI, CT scan	Mature cystic teratoma	4x4	Anterior-middle mediastinum	Unilateral (right)	Uneventful	(17)
Rüffer	2012	1	16	Female	Emesis, diarrhea, fever	X-ray, MRI, CT scan	Mature cystic teratoma	8x8	Anterior mediastinum	Unilateral (right)	Uneventful	(18)
Chang	2008	1	52	Female	Asymptomatic	CT scan	Mature cystic teratoma	6.5x5.3	The right-sided paratracheal mediastinum	Unilateral (right)	Recurrence	(19)
Furukawa	1994	1	23	Female	Asymptomatic	X-ray, CT scan	Mature cystic teratoma	7x5	Anterior mediastinum	Unilateral (right)	Uneventful	(20)

CT scan, computed tomography scan; MRI, magnetic resonance imaging; VATS, video-assisted thoracic surgery; Echo, echocardiogram; TMT, teratoma with malignant transformation.

## Data Availability

The data generated in the present study may be requested from the corresponding author.
